# Supplementary material for: Ancestral protein reconstruction reveals evolutionary events governing variation in Dicer helicase function
Source: eLife. 2023 Apr 17;12:e85120. doi: 10.7554/eLife.85120 (PMC10159624; doi:10.7554/eLife.85120)
Supplement: Figure 1—figure supplement 4—source data 1. [file elife-85120-fig1-figsupp4-data1.zip › Figure 1-figure supplement 4 - Source data 1.pdf]

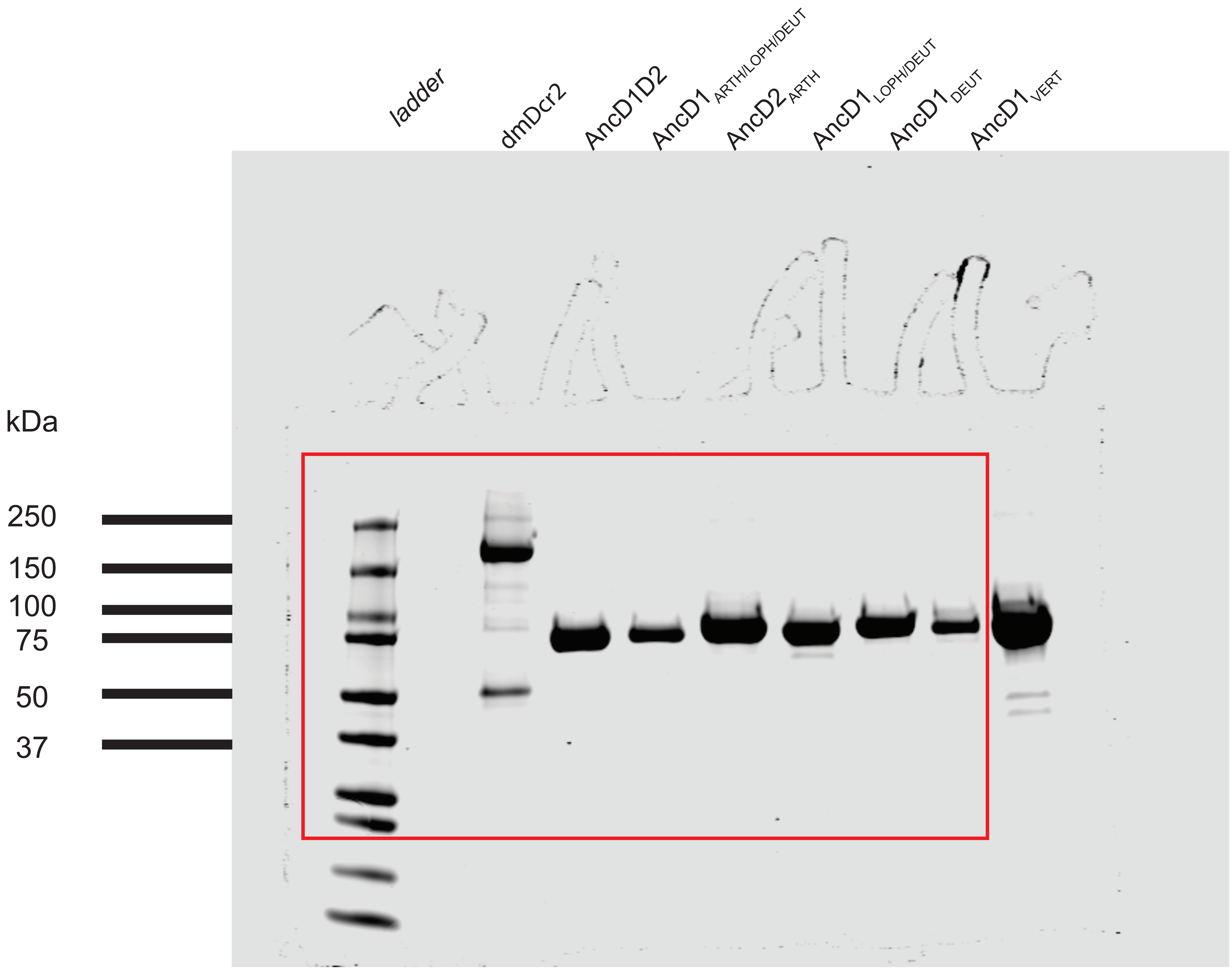

Figure 1-figure supplement 4 - source data 1: Original digital image of SDS-PAGE scanned gel used in B. Region used indicated in red rectangle.
