# Supplementary figures and images for: Ancestral protein reconstruction reveals evolutionary events governing variation in Dicer helicase function
Source: eLife. 2023 Apr 17;12:e85120. doi: 10.7554/eLife.85120 (PMC10159624; doi:10.7554/eLife.85120)

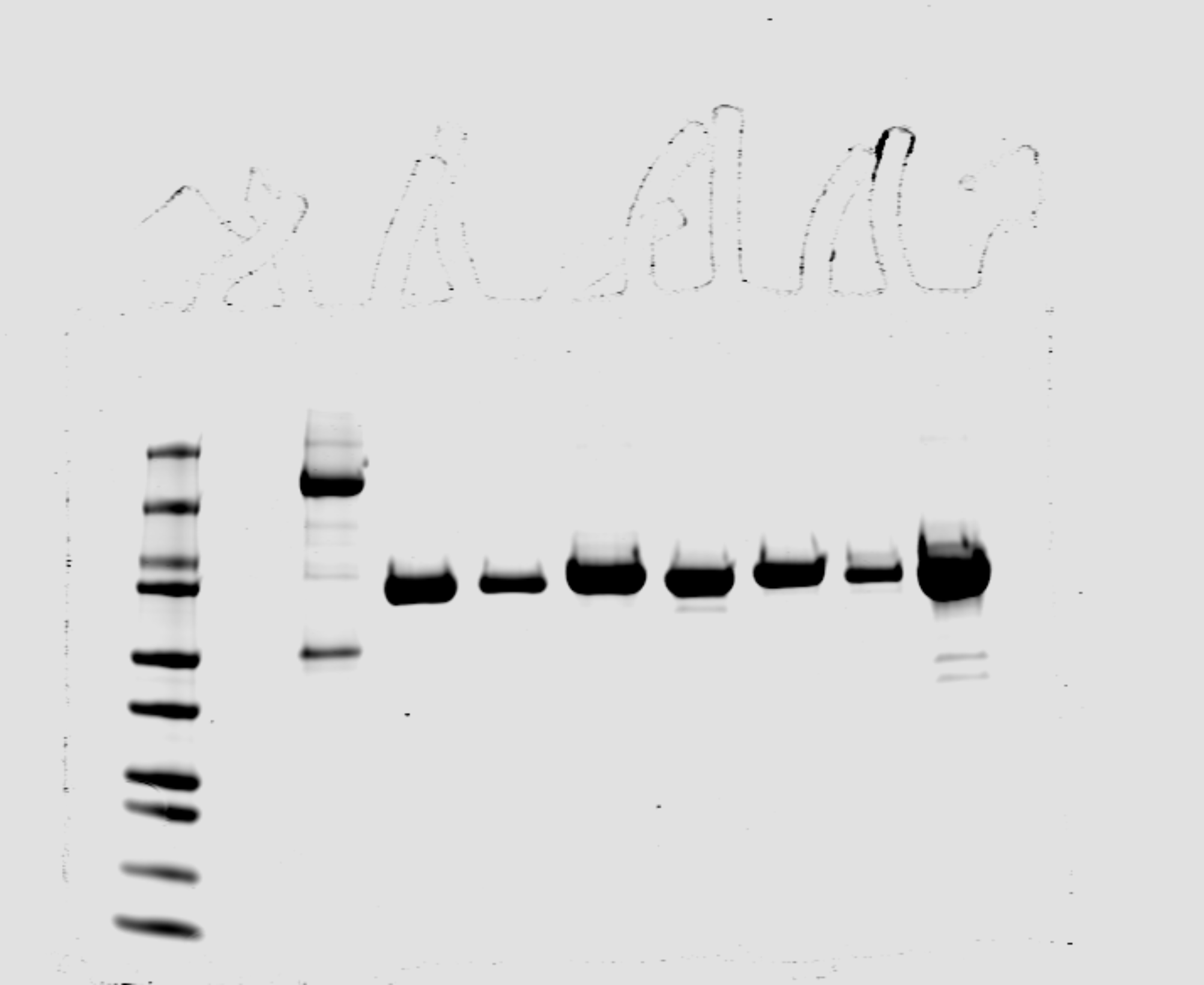

Supplement: Figure 1—figure supplement 4—source data 1. [file elife-85120-fig1-figsupp4-data1.zip › FIGURE 1-FIGURE SUPPLEMENT 4 - SOURCE DATA 1.tif]

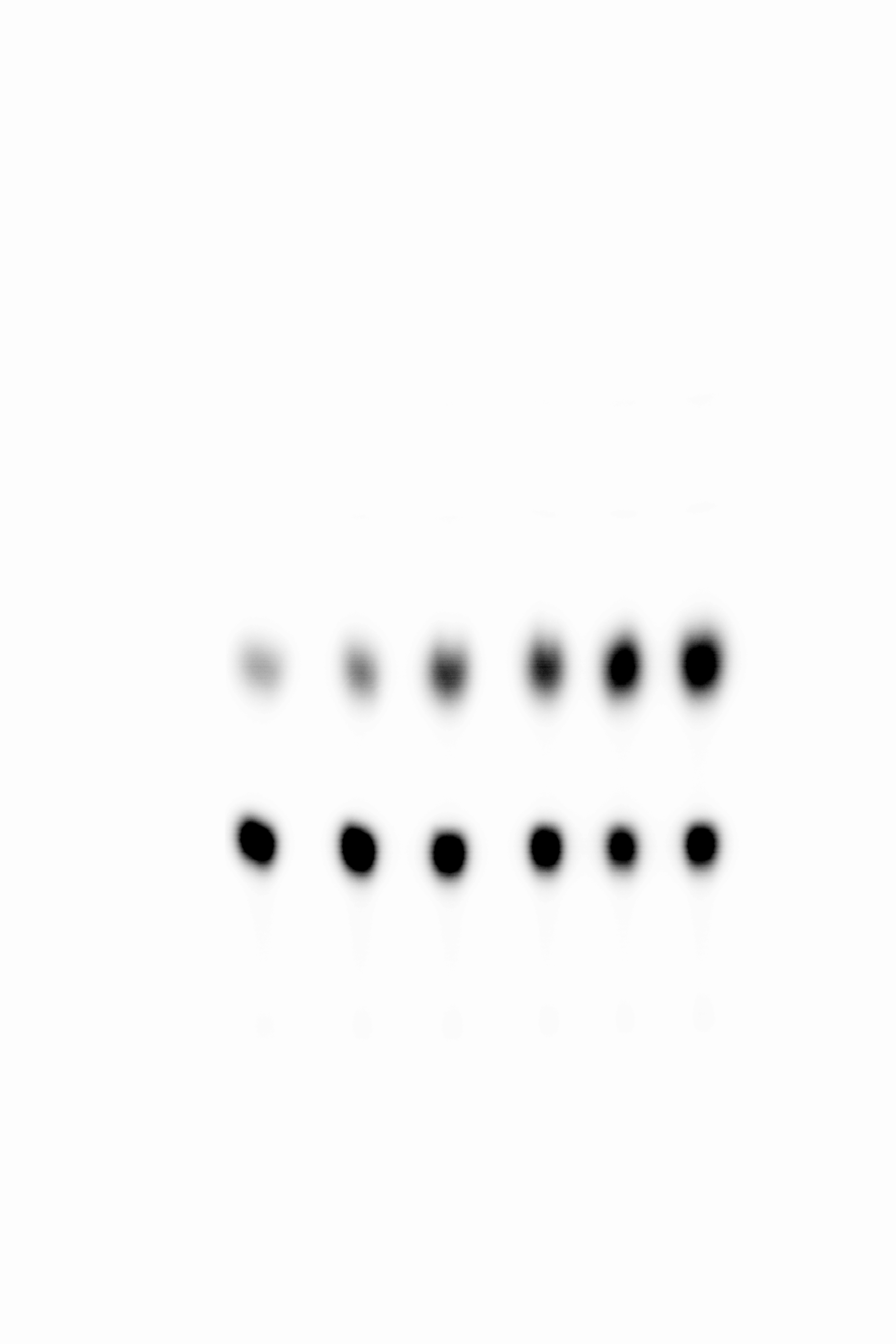

Supplement: Figure 2—source data 3. [file elife-85120-fig2-data3.zip › FIGURE 2 - SOURCE DATA 3 ANCD2ARTH NO RNA.bmp]

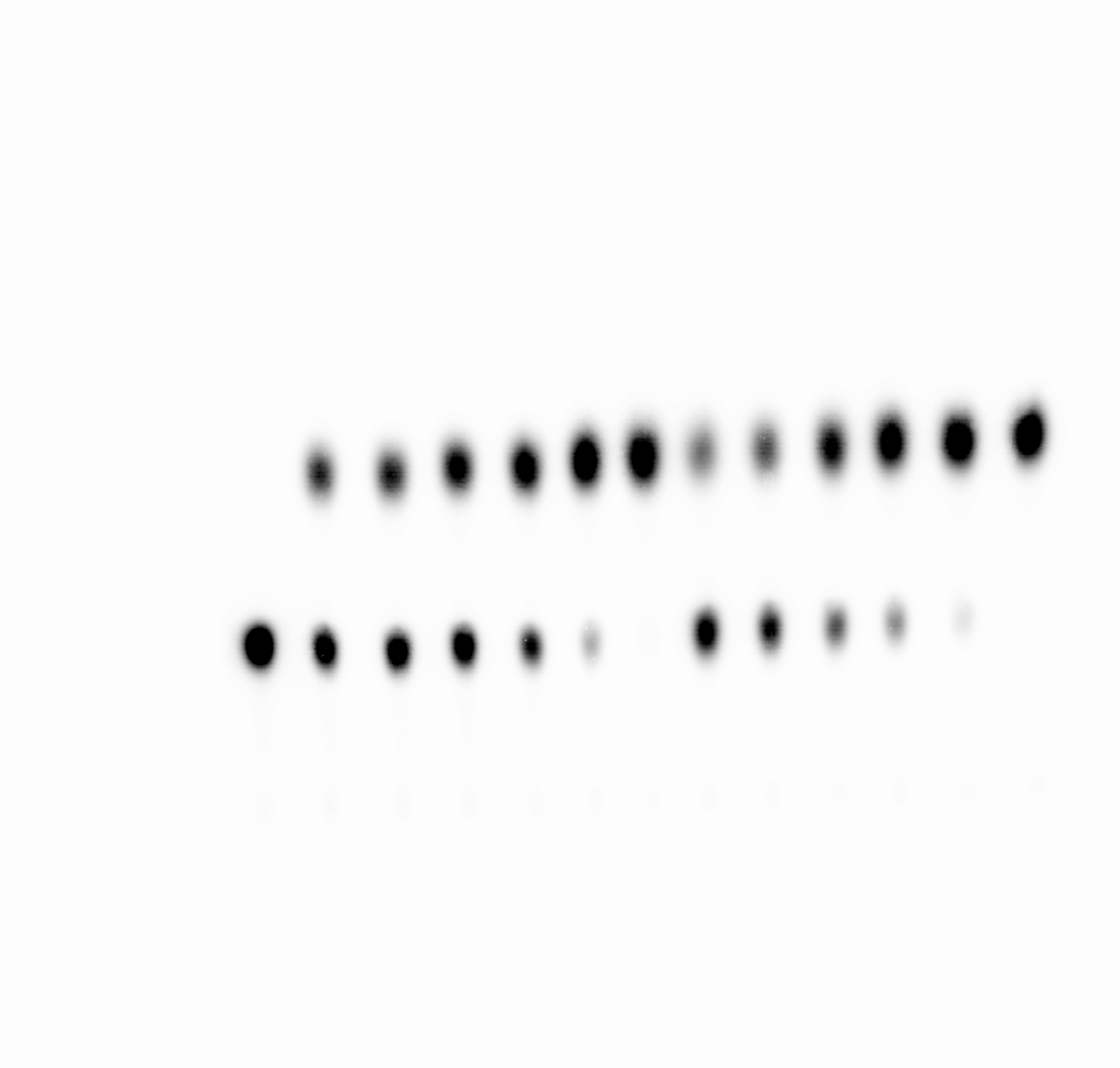

Supplement: Figure 2—source data 4. [file elife-85120-fig2-data4.zip › FIGURE 2 - SOURCE DATA 4 ANCD2ARTH BLT 3'OVR.bmp]

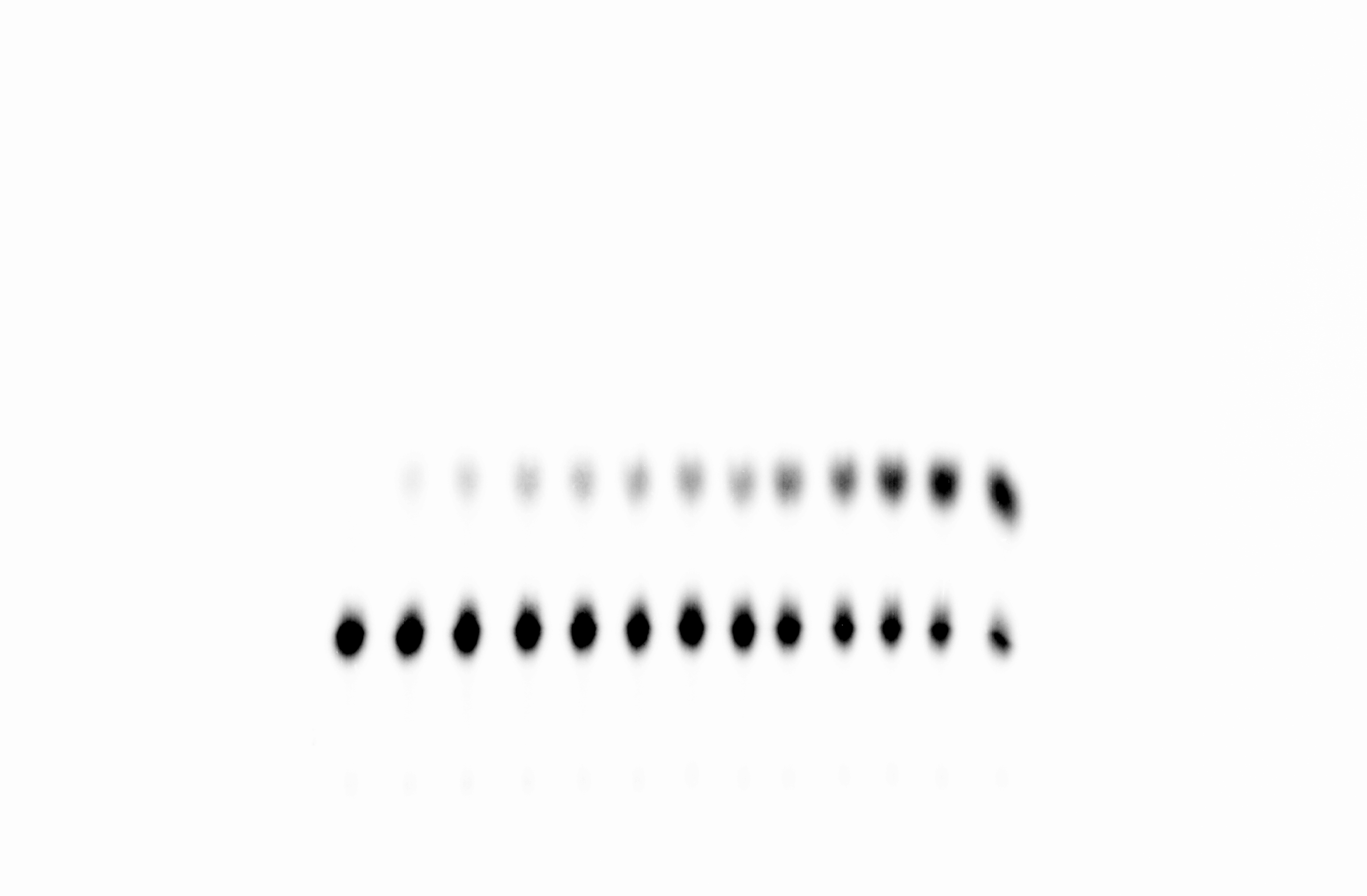

Supplement: Figure 2—source data 5. [file elife-85120-fig2-data5.zip › FIGURE 2 - SOURCE DATA 5 ANCD1DEUT NO RNA.bmp]

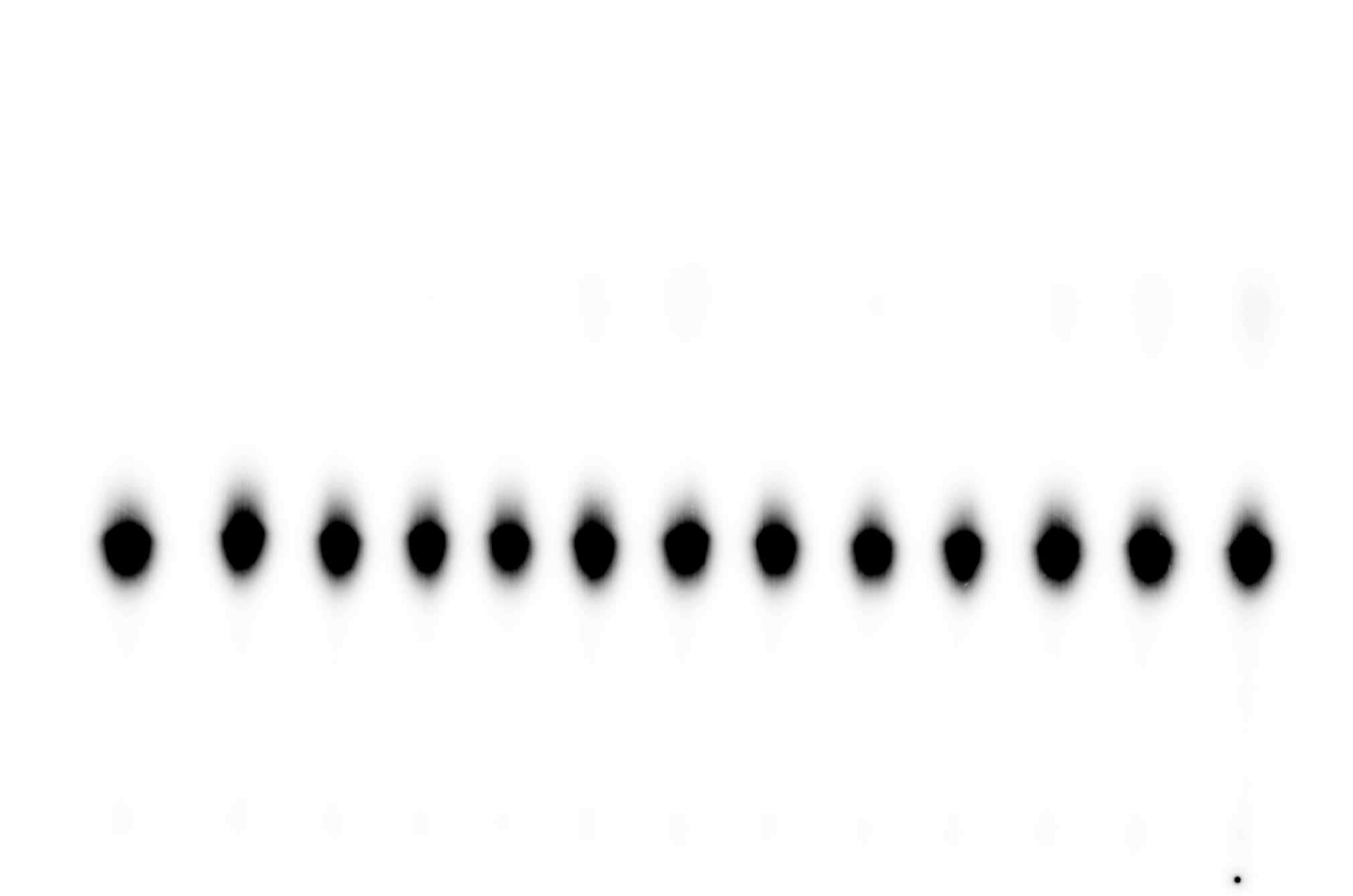

Supplement: Figure 2—source data 7. [file elife-85120-fig2-data7.zip › FIGURE 2 - SOURCE DATA 7 ANCD1VERT NO RNA.bmp]

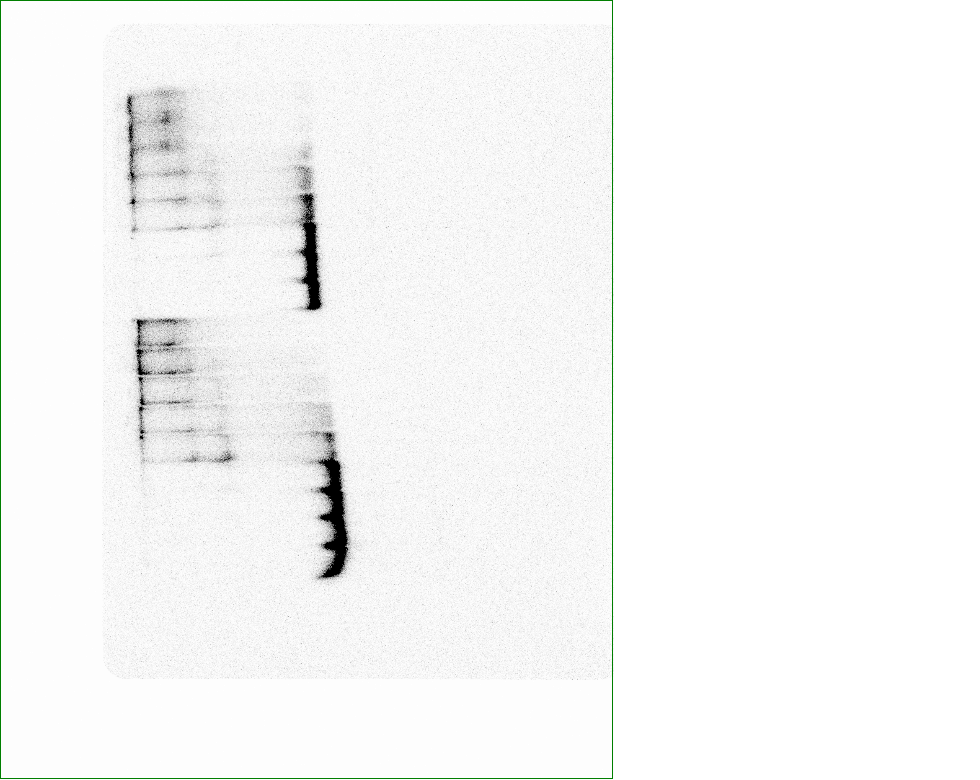

Supplement: Figure 3—source data 1. [file elife-85120-fig3-data1.zip › FIGURE 3 - SOURCE DATA 1 ANCD1D2 BLT.bmp]

# AncD1D2

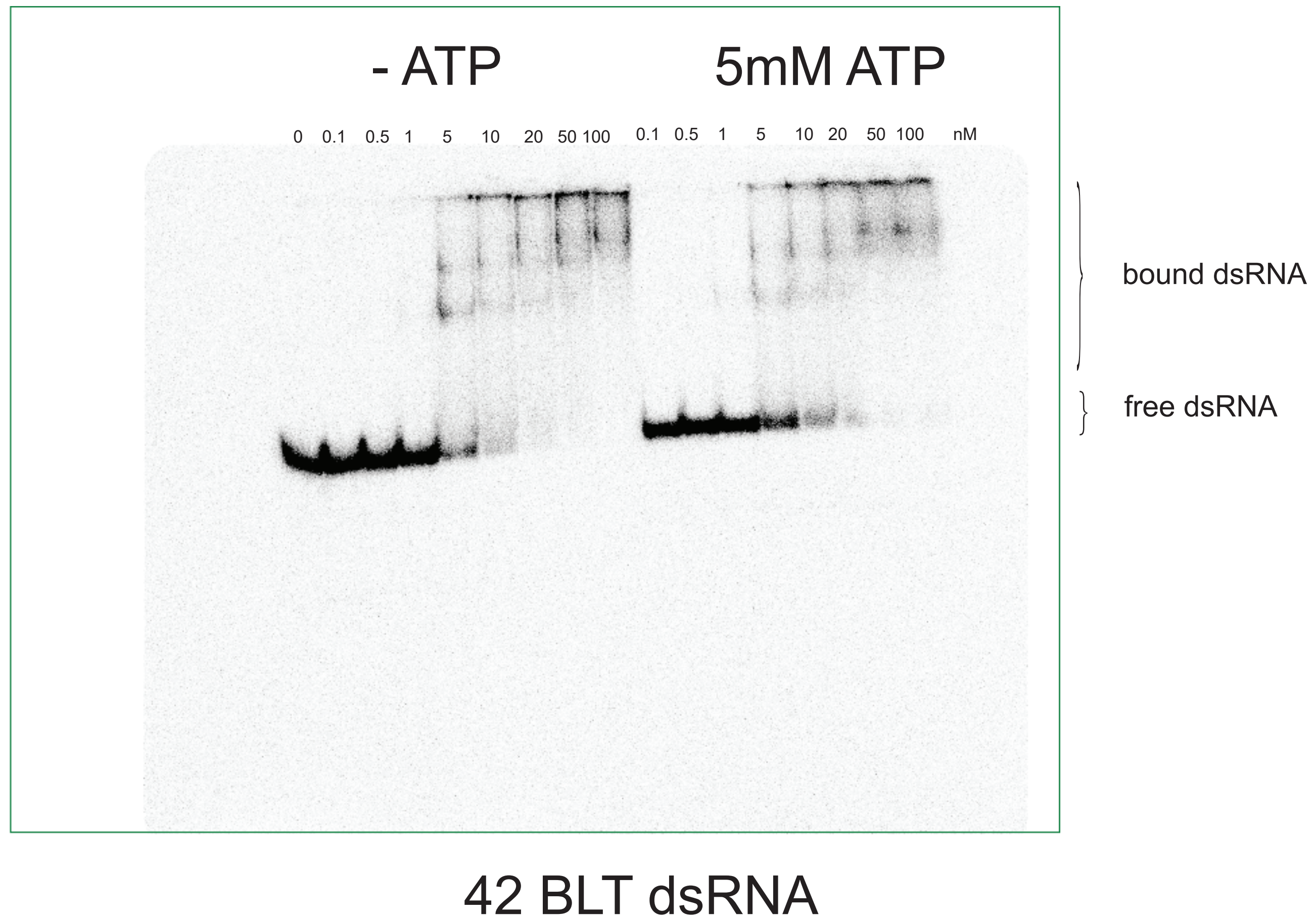

Figure 3 - source data 1: Original digital image of phosphorimager scan used in Figure 3B.

Supplement: Figure 3—source data 1. [file elife-85120-fig3-data1.zip › FIGURE 3 - SOURCE DATA 1 ANCD1D2 BLT.pdf]

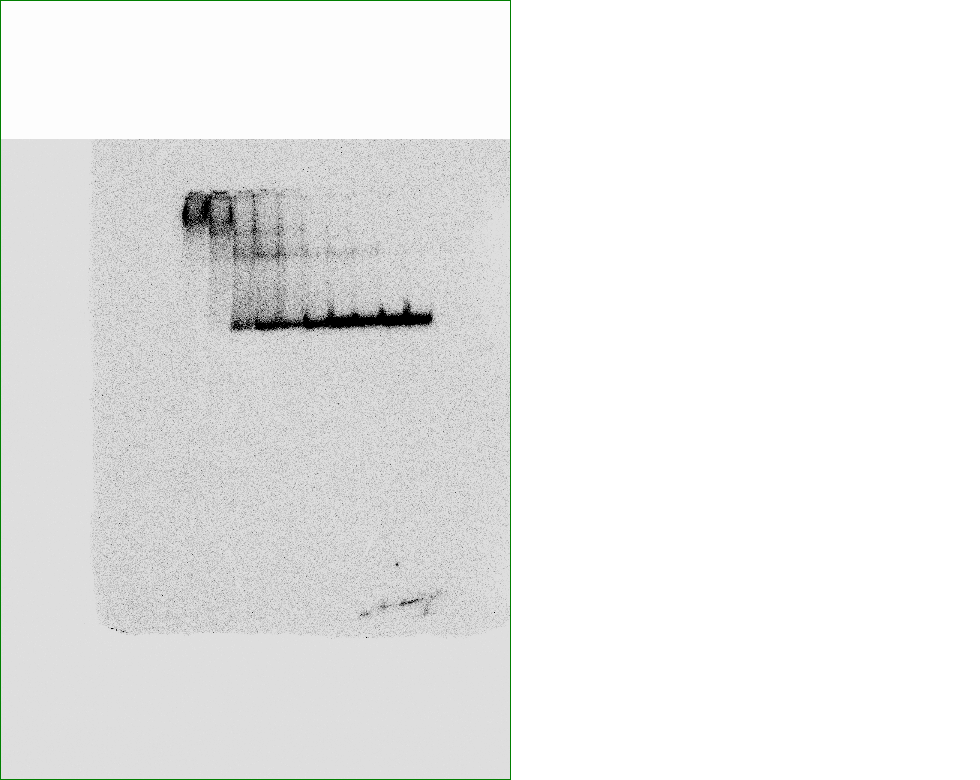

Supplement: Figure 3—source data 2. [file elife-85120-fig3-data2.zip › FIGURE 3 - SOURCE DATA 2 ANCD1D2 3'OVR NO ATP.bmp]

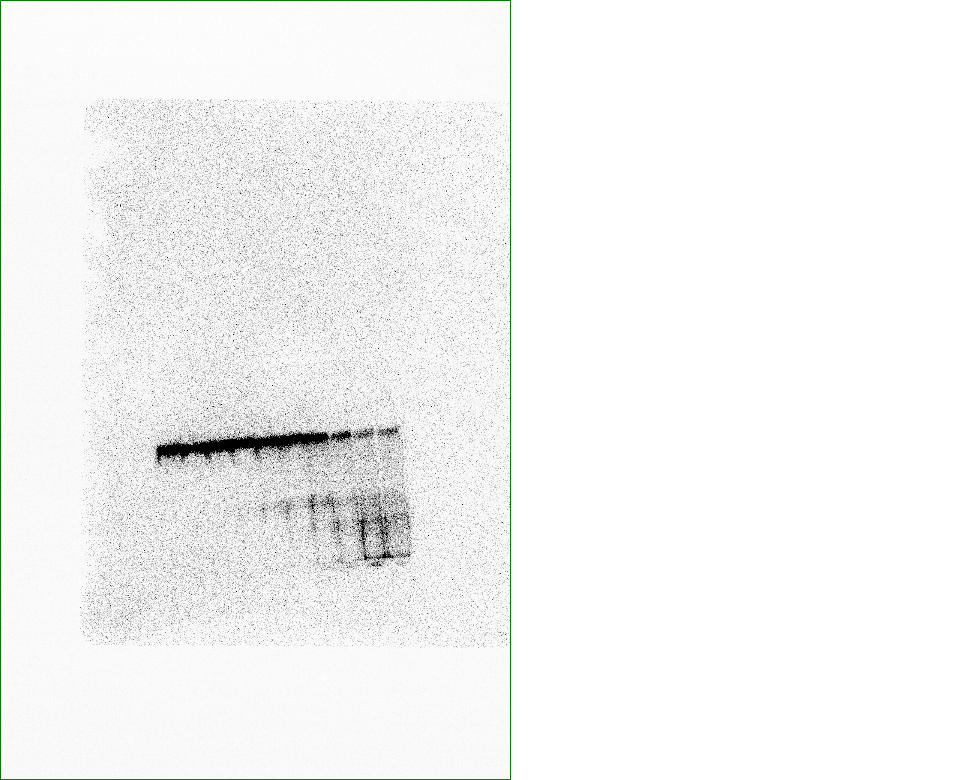

Supplement: Figure 3—source data 3. [file elife-85120-fig3-data3.zip › FIGURE 3 - SOURCE DATA 3 ANCD1D2 3'OVR PLUS ATP.bmp]

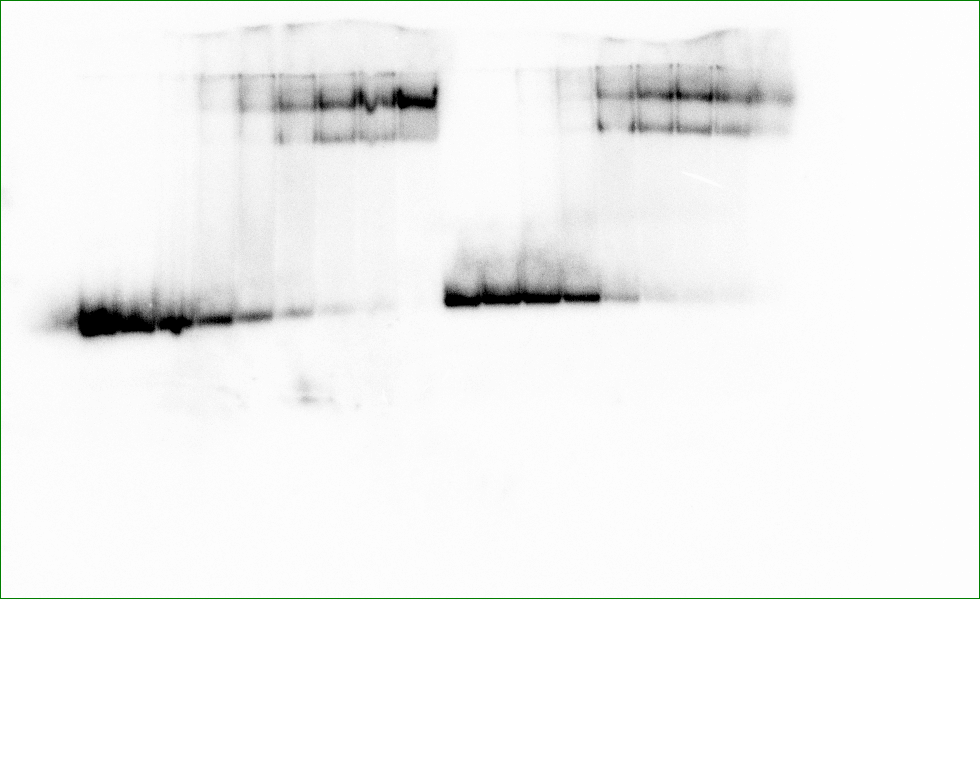

Supplement: Figure 3—source data 4. [file elife-85120-fig3-data4.zip › FIGURE 3 - SOURCE DATA 4 ANCD1DEUT BLT.bmp]

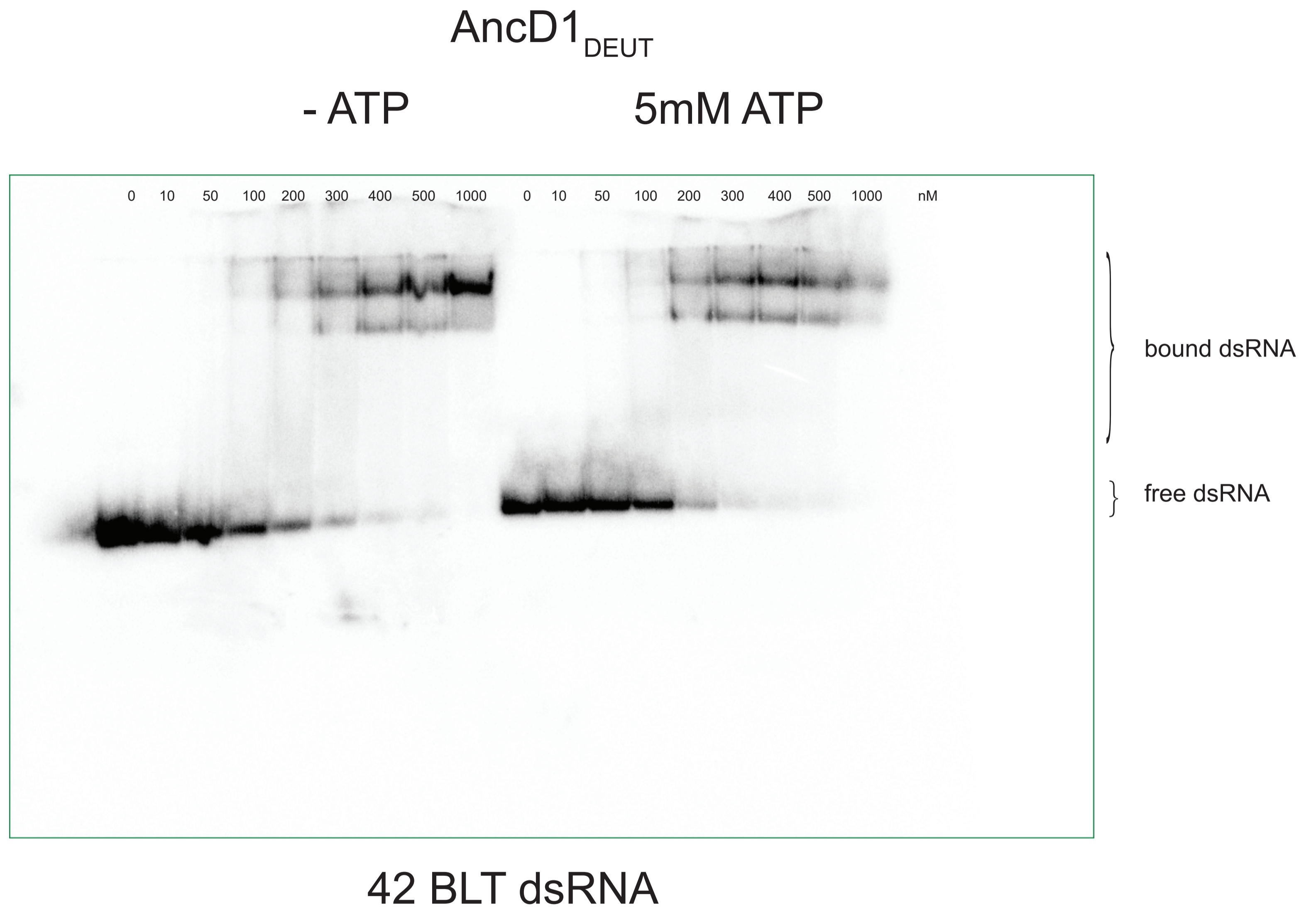

Figure 3 - source data 4: Original digital image of phosphorimager scan used in Figure 3D.

Supplement: Figure 3—source data 4. [file elife-85120-fig3-data4.zip › FIGURE 3 - SOURCE DATA 4 ANCD1DEUT BLT.pdf]

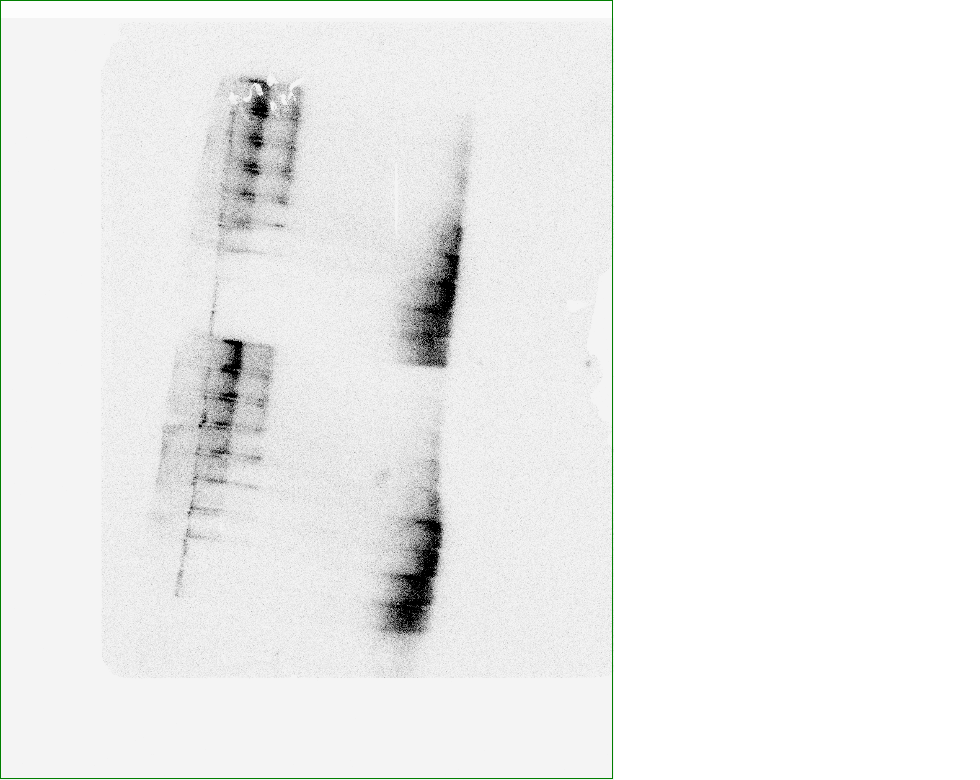

Supplement: Figure 3—source data 5. [file elife-85120-fig3-data5.zip › FIGURE 3 - SOURCE DATA 5 ANCD1DEUT 3'OVR.bmp]

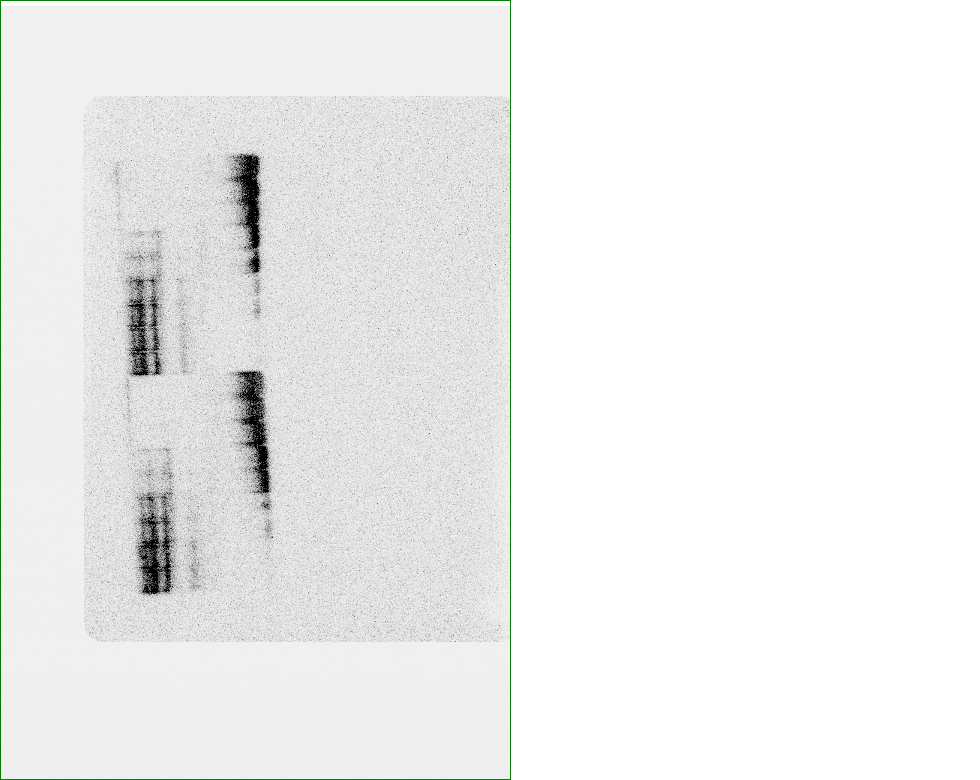

Supplement: Figure 3—source data 6. [file elife-85120-fig3-data6.zip › FIGURE 3 - SOURCE DATA 6 ANCD1VERT BLT.bmp]

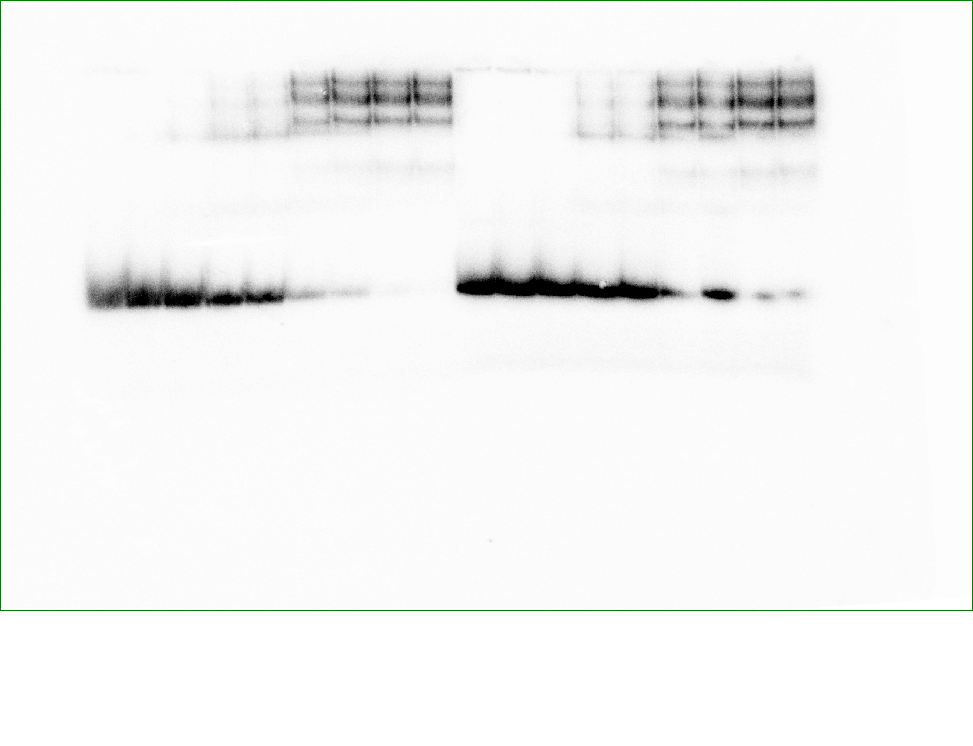

Supplement: Figure 3—source data 7. [file elife-85120-fig3-data7.zip › FIGURE 3 - SOURCE DATA 7 ANCD1VERT BLT 3'OVR.bmp]

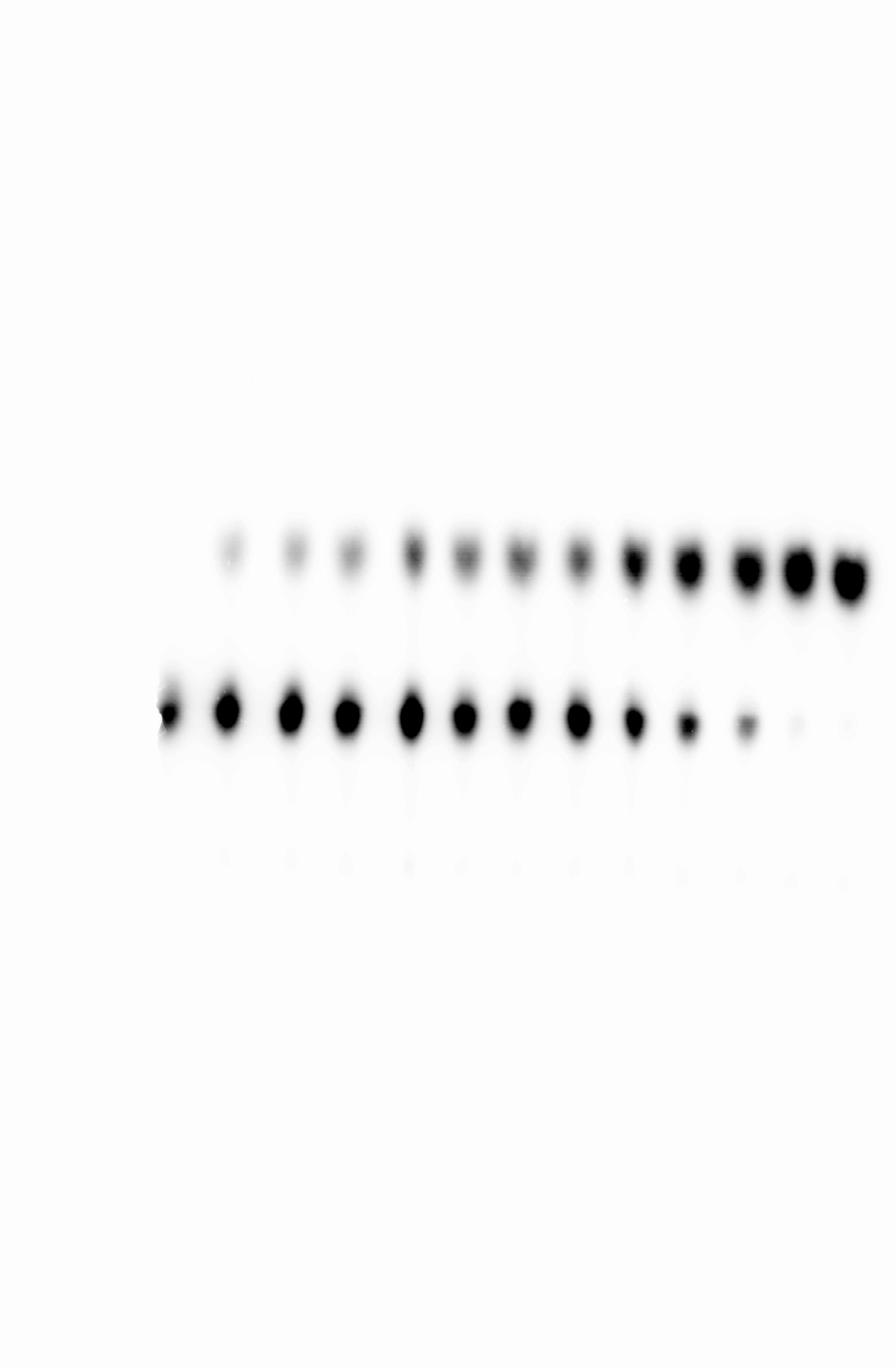

Supplement: Figure 4—figure supplement 3—source data 3. [file elife-85120-fig4-figsupp3-data3.zip › FIGURE 4-FIGURE SUPPLEMENT 3 - SOURCE DATA 3.bmp]

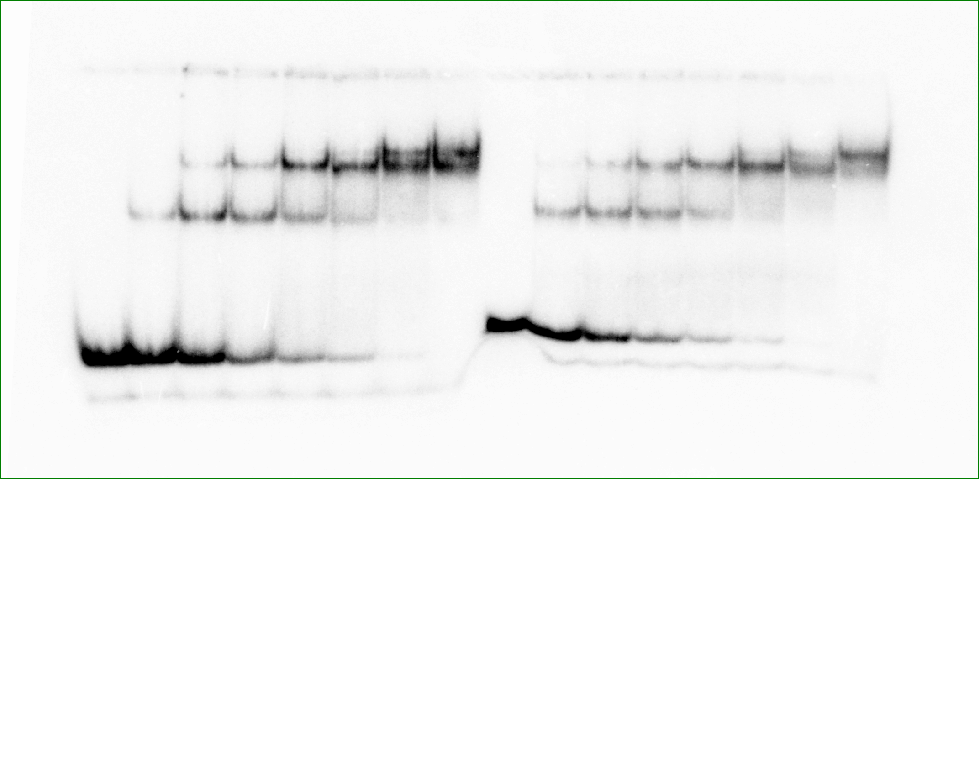

Supplement: Figure 4—figure supplement 4—source data 1. [file elife-85120-fig4-figsupp4-data1.zip › FIGURE 4-FIGURE SUPPLEMENT 4 - SOURCE DATA 1.bmp]

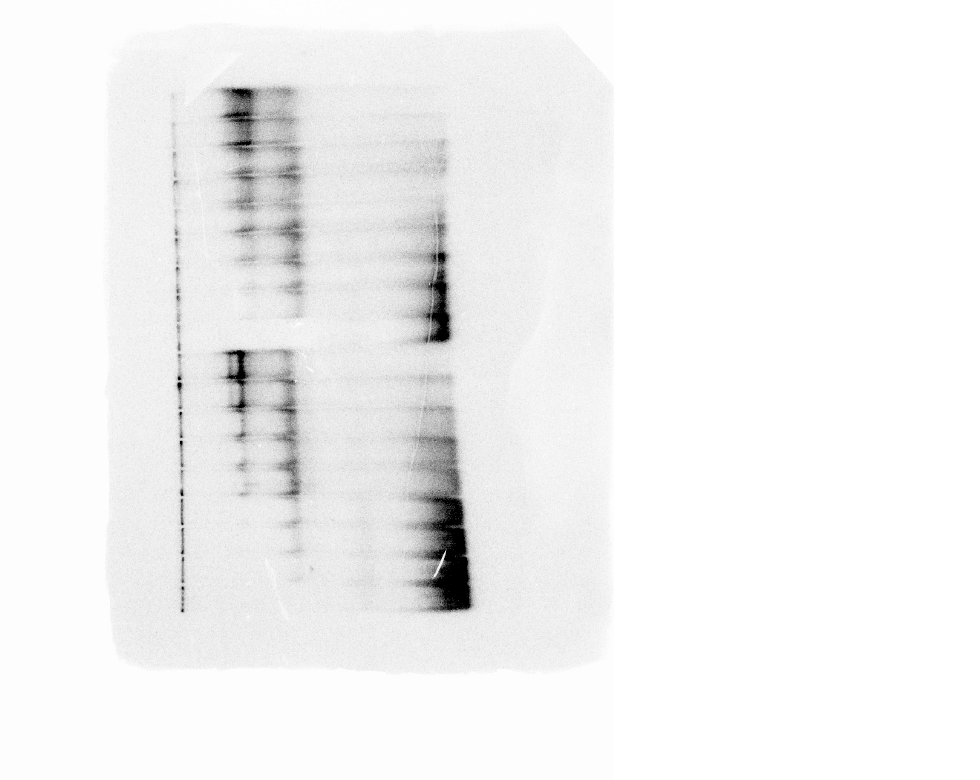

Supplement: Figure 4—figure supplement 4—source data 2. [file elife-85120-fig4-figsupp4-data2.zip › FIGURE 4-FIGURE SUPPLEMENT 4 - SOURCE DATA 2.bmp]

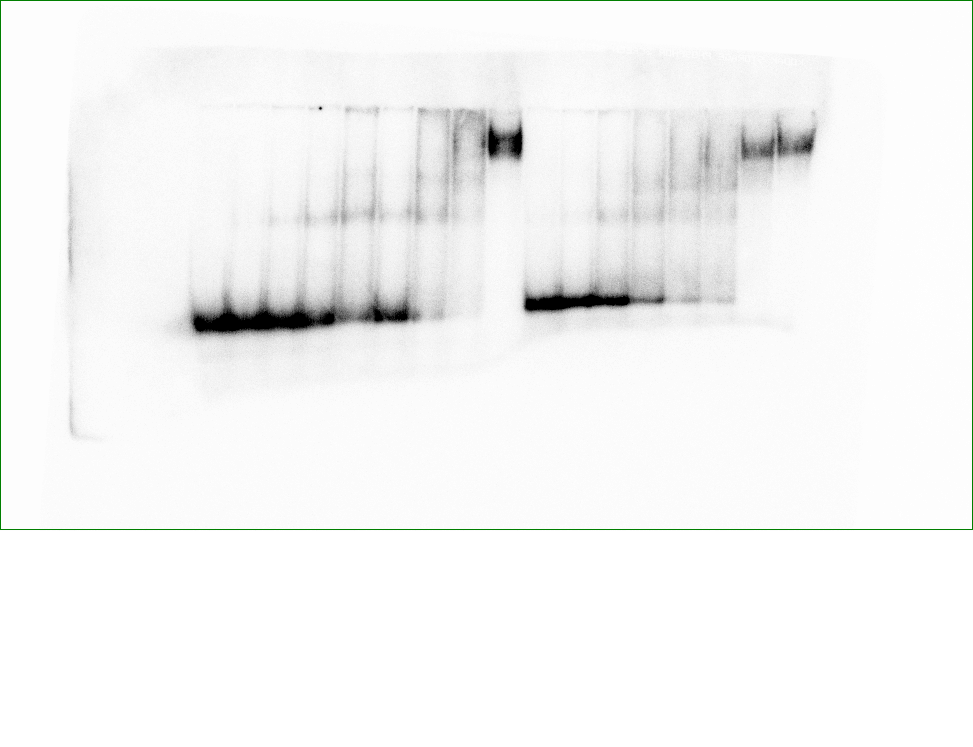

Supplement: Figure 4—figure supplement 4—source data 3. [file elife-85120-fig4-figsupp4-data3.zip › FIGURE 4-FIGURE SUPPLEMENT 4 - SOURCE DATA 3.bmp]

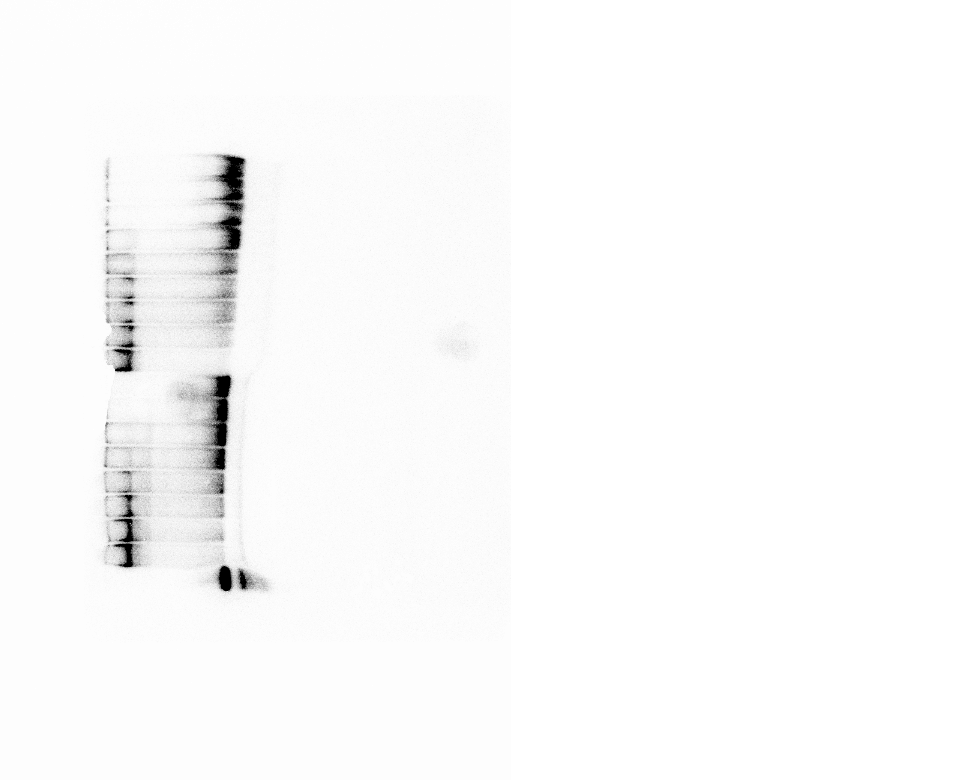

Supplement: Figure 4—figure supplement 4—source data 4. [file elife-85120-fig4-figsupp4-data4.zip › FIGURE 4-FIGURE SUPPLEMENT 4 - SOURCE DATA 4.bmp]
