# Supplementary material for: Ancestral protein reconstruction reveals evolutionary events governing variation in Dicer helicase function
Source: eLife. 2023 Apr 17;12:e85120. doi: 10.7554/eLife.85120 (PMC10159624; doi:10.7554/eLife.85120)
Supplement: Figure 3—source data 3. [file elife-85120-fig3-data3.zip › FIGURE 3 - SOURCE DATA 3 ANCD1D2 3'OVR PLUS ATP.pdf]

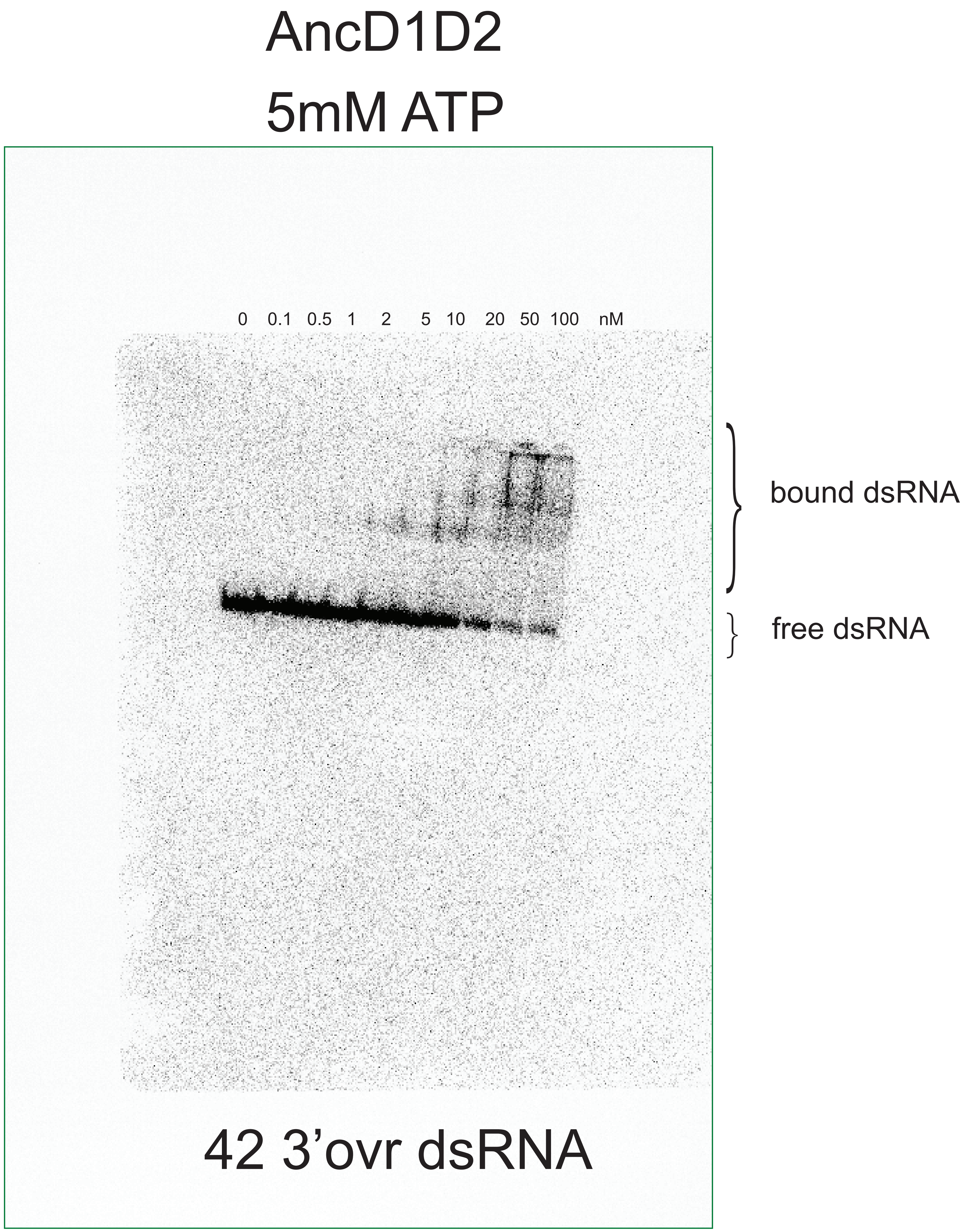

Figure 3 - source data 3: Original digital image of phosphoimager scan used in Figure 3C, right panel.
