# Supplementary material for: Ancestral protein reconstruction reveals evolutionary events governing variation in Dicer helicase function
Source: eLife. 2023 Apr 17;12:e85120. doi: 10.7554/eLife.85120 (PMC10159624; doi:10.7554/eLife.85120)
Supplement: Figure 3—source data 6. [file elife-85120-fig3-data6.zip › FIGURE 3 - SOURCE DATA 6 ANCD1VERT BLT.pdf]

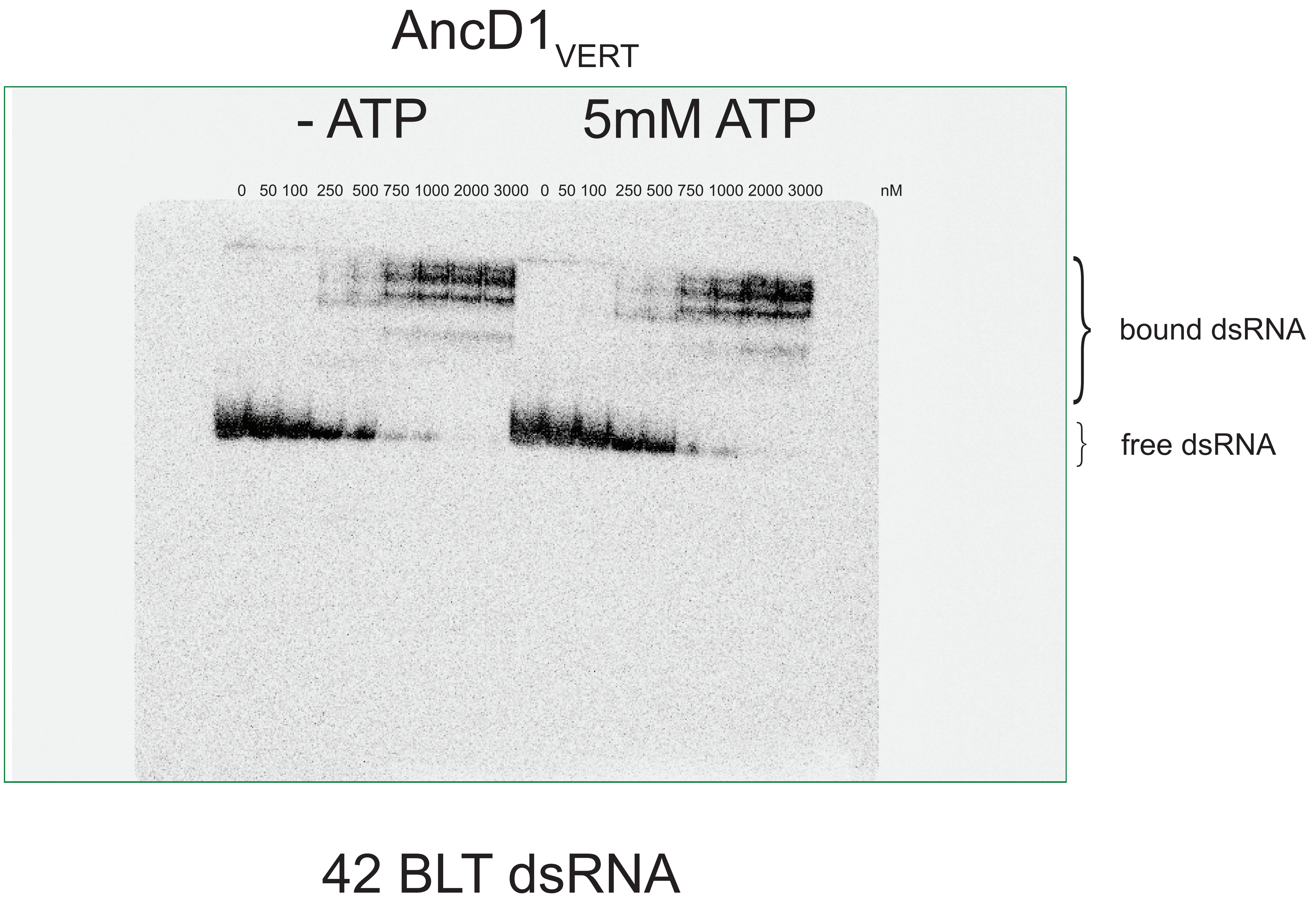


Figure 3 - source data 6: Original digital image of phosphoimager scan used in Figure 3F.
