# Supplementary material for: Ancestral protein reconstruction reveals evolutionary events governing variation in Dicer helicase function
Source: eLife. 2023 Apr 17;12:e85120. doi: 10.7554/eLife.85120 (PMC10159624; doi:10.7554/eLife.85120)

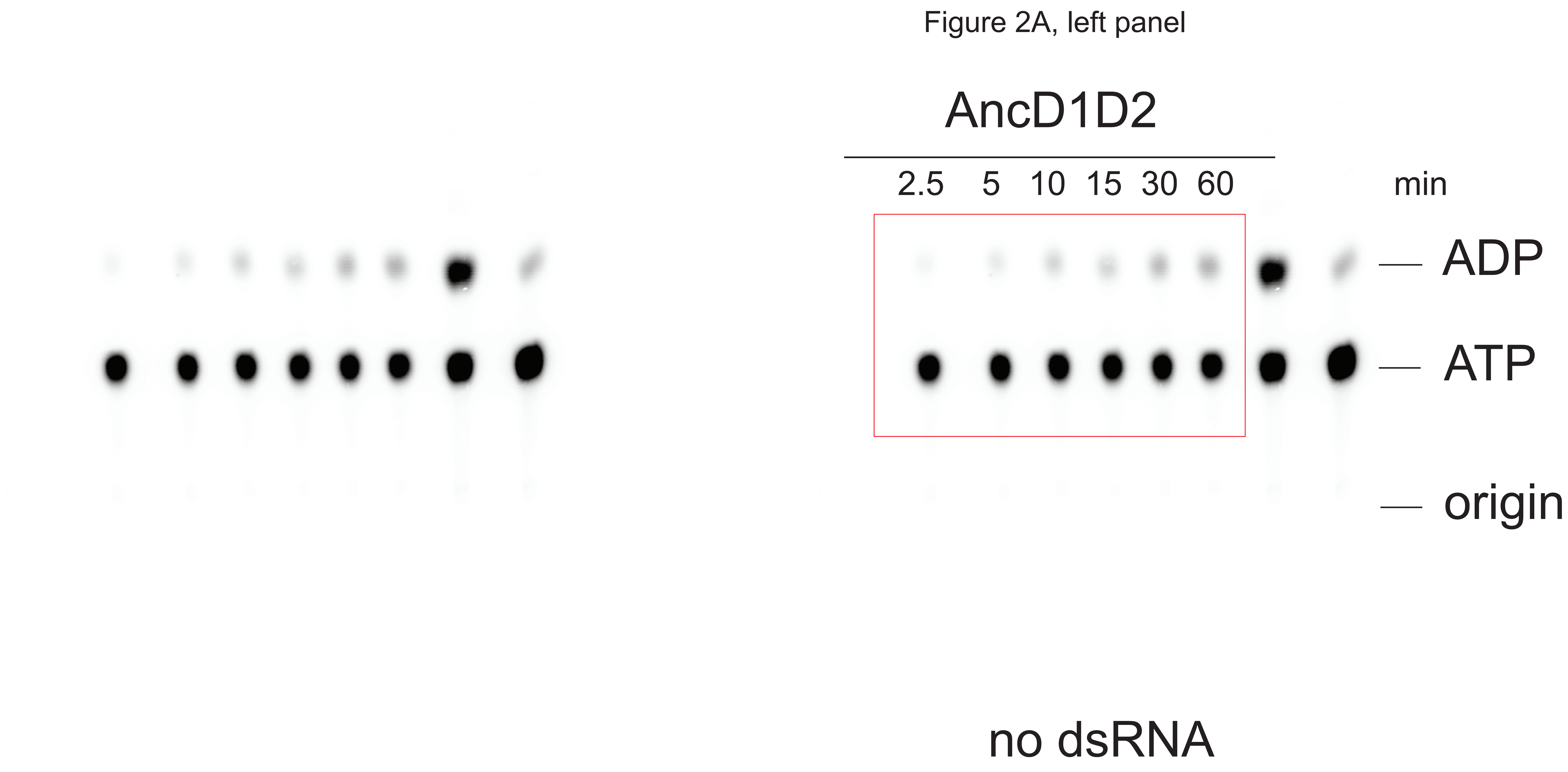

Figure 2 - source Data 1: Duplcate digital images of Thin Layer Chromatography plate. Image on the right shows region of the plate used in Figure 2A, left panel.

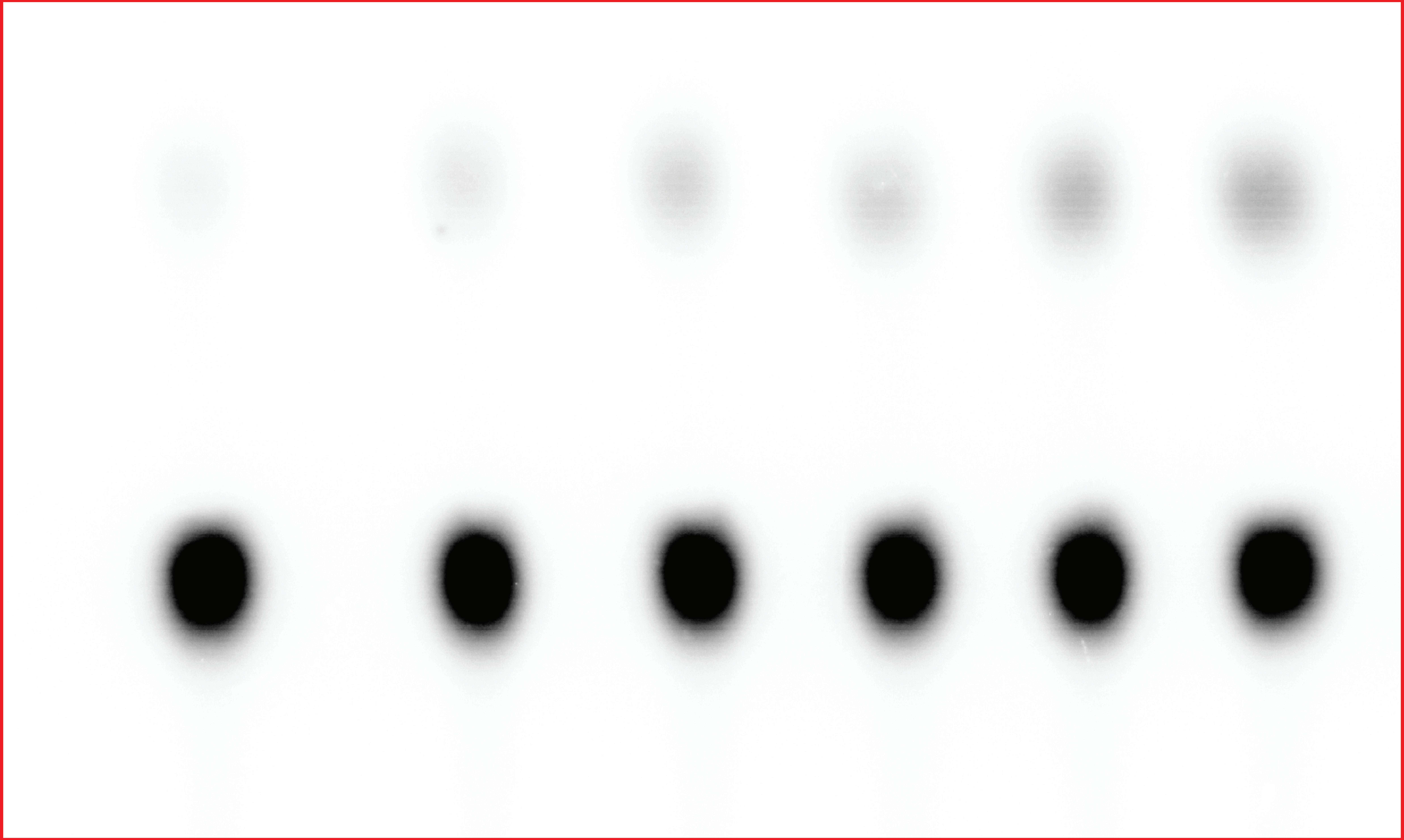

Supplement: Figure 2—source data 1. [file FIGURE 2 - SOURCE DATA 1 ANCD1D2 NO RNA.pdf › FIGURE 2 - SOURCE DATA 1 ANCD1D2 NO RNA.pdf]
