# Supplementary material for: Ancestral protein reconstruction reveals evolutionary events governing variation in Dicer helicase function
Source: eLife. 2023 Apr 17;12:e85120. doi: 10.7554/eLife.85120 (PMC10159624; doi:10.7554/eLife.85120)
Supplement: Figure 2—source data 2. [file FIGURE 2 - SOURCE DATA 2 ANCD1D2 BLT 3'OVR.pdf › FIGURE 2 - SOURCE DATA 2 ANCD1D2 BLT 3'OVR.pdf]

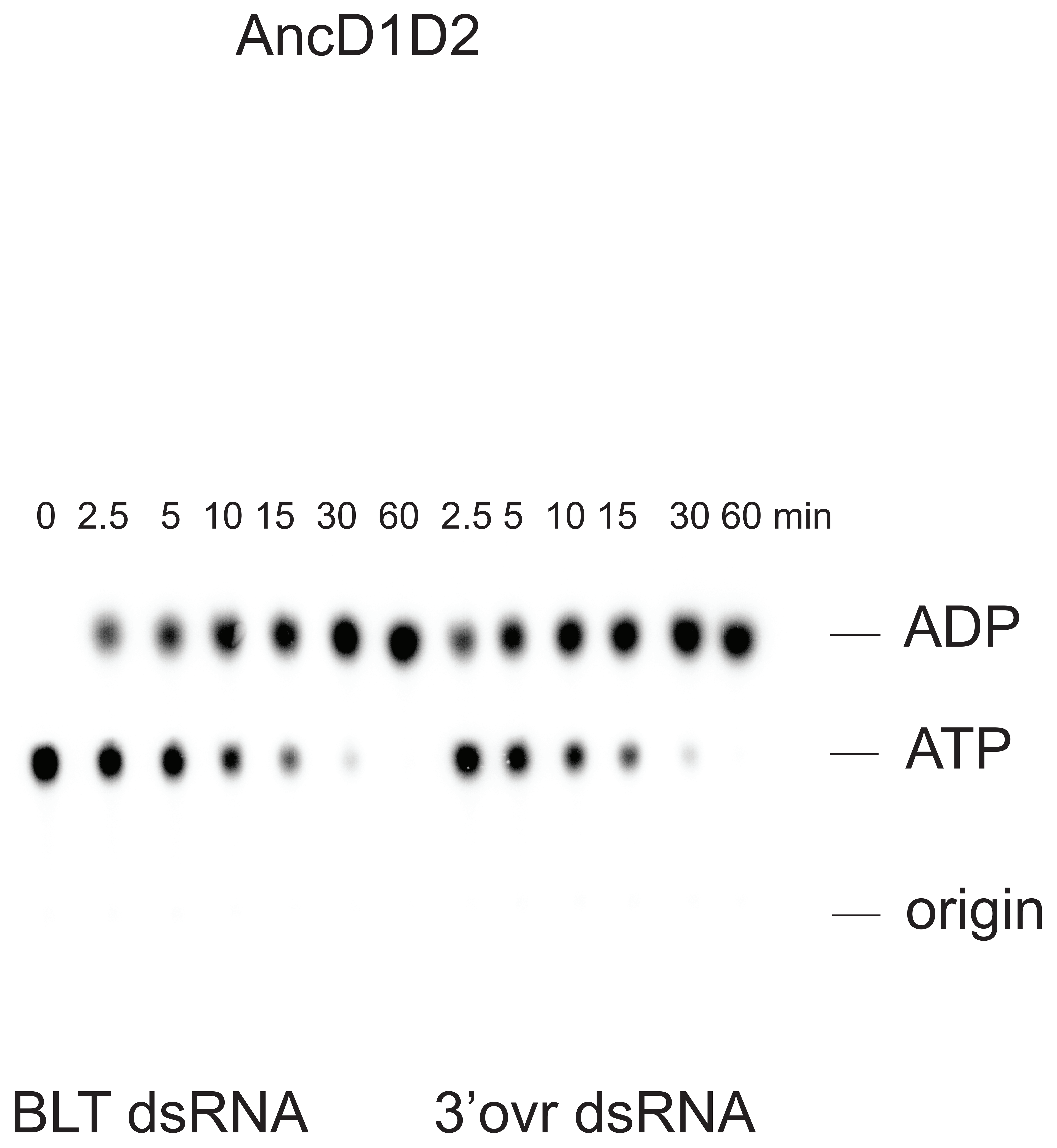

Figure 2 - source Data 2: Original digital image of Thin Layer Chromatography plate used in Figure 2A, right panel.
