# Supplementary material for: Ancestral protein reconstruction reveals evolutionary events governing variation in Dicer helicase function
Source: eLife. 2023 Apr 17;12:e85120. doi: 10.7554/eLife.85120 (PMC10159624; doi:10.7554/eLife.85120)
Supplement: Figure 2—source data 5. [file FIGURE 2 - SOURCE DATA 5 ANCD1DEUT NO DSRNA.pdf › FIGURE 2 - SOURCE DATA 5 ANCD1DEUT NO DSRNA.pdf]

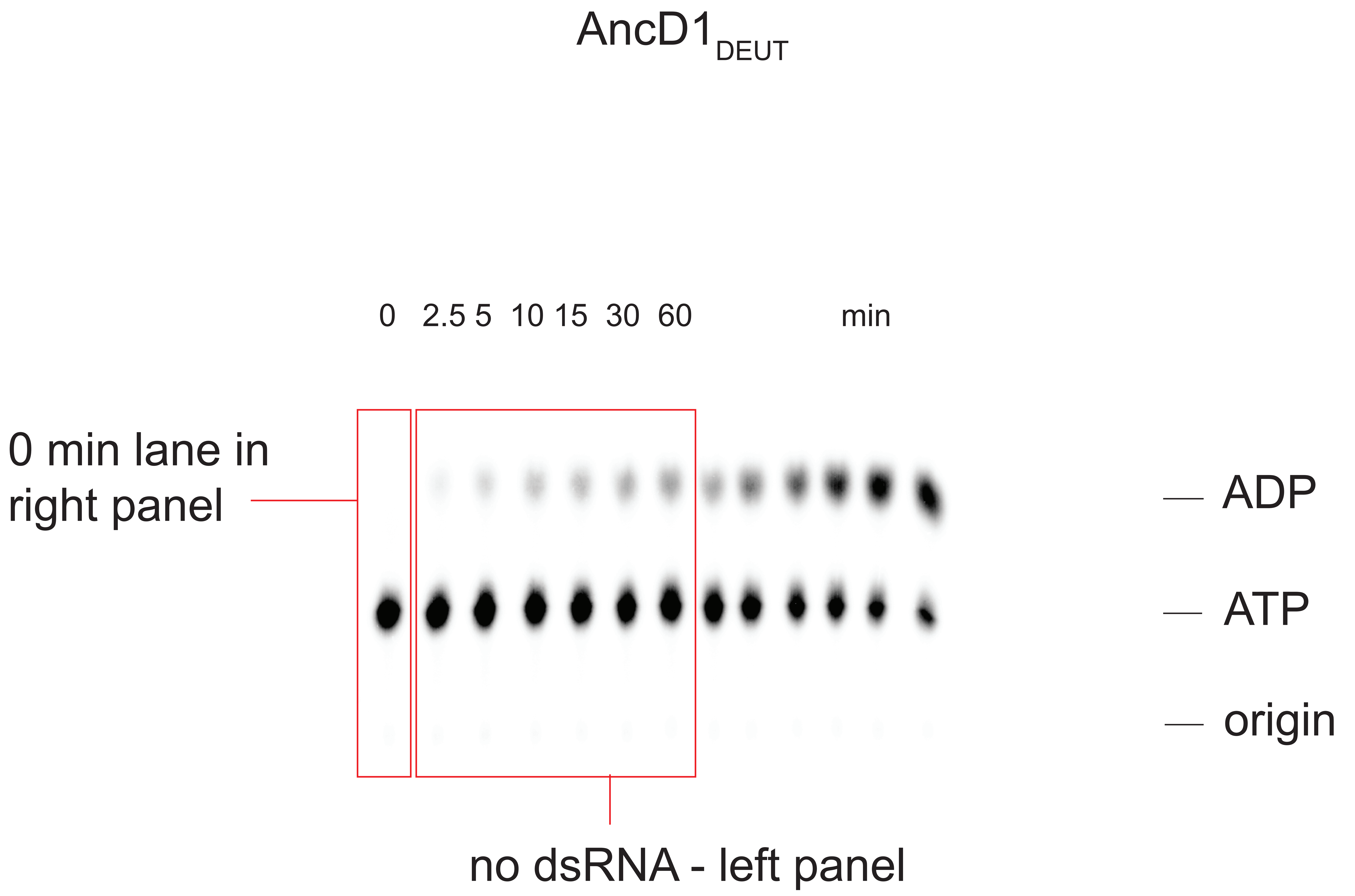

Figure 2 - source data 5: Original digital image of Thin Layer Chromatography plate. Regions used in Figure 2C indicated by red rectangles.
