# Supplementary material for: Ancestral protein reconstruction reveals evolutionary events governing variation in Dicer helicase function
Source: eLife. 2023 Apr 17;12:e85120. doi: 10.7554/eLife.85120 (PMC10159624; doi:10.7554/eLife.85120)
Supplement: Figure 2—source data 8. [file FIGURE 2 - SOURCE DATA 8 ANCD1VERT BLT and 3'OVR.pdf › FIGURE 2 - SOURCE DATA 8 ANCD1VERT BLT and 3'OVR.pdf]

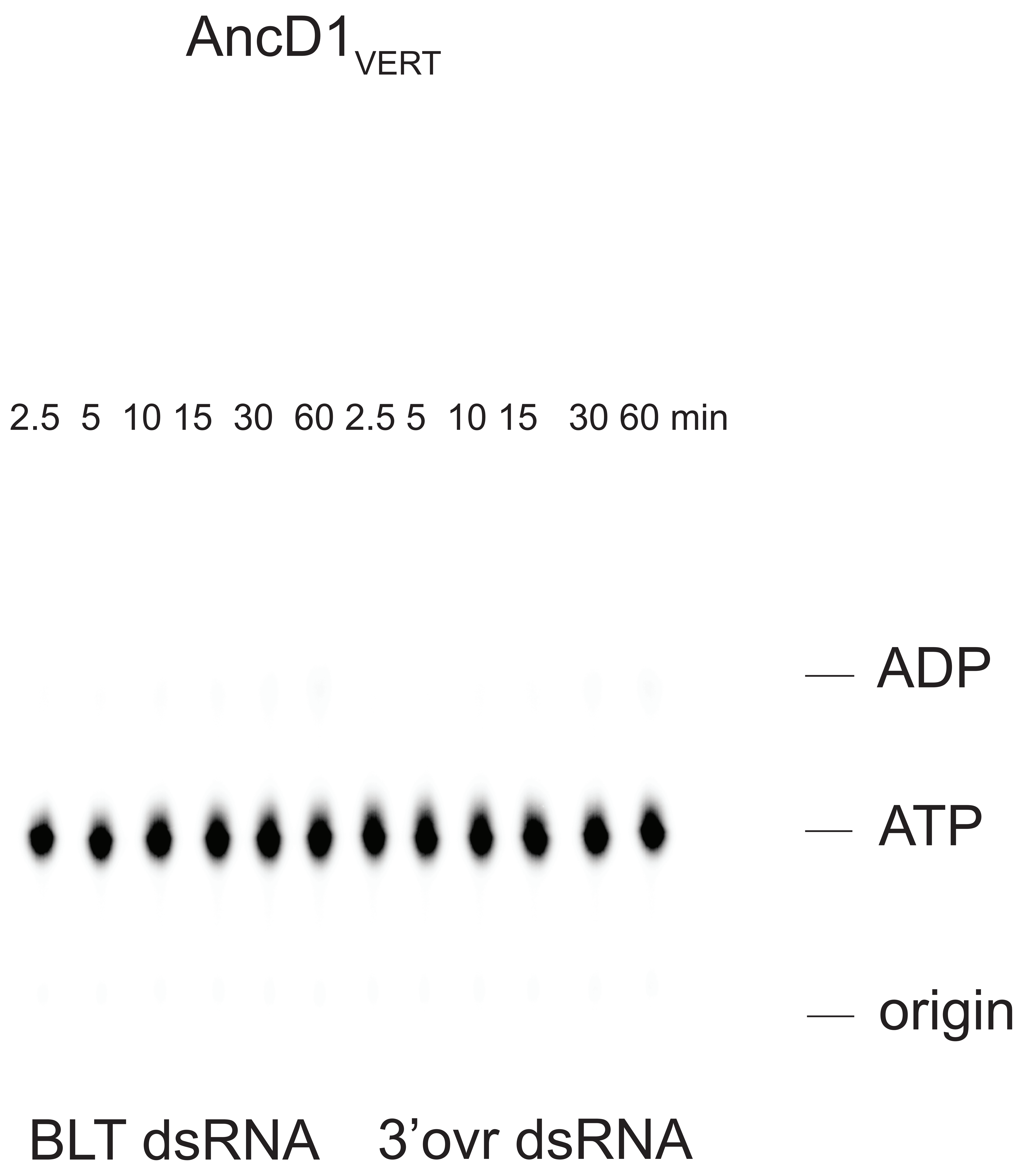

Figure 2 - source data 8: Original digital image of Thin Layer Chromatography plate used in Figure 2D, right panel.
