# Supplementary material for: Ancestral protein reconstruction reveals evolutionary events governing variation in Dicer helicase function
Source: eLife. 2023 Apr 17;12:e85120. doi: 10.7554/eLife.85120 (PMC10159624; doi:10.7554/eLife.85120)
Supplement: Figure 4—figure supplement 3—source data 1. [file FIGURE 4-FIGURE SUPPLEMENT 3 - SOURCE DATA 1.pdf › FIGURE 4-FIGURE SUPPLEMENT 3 - SOURCE DATA 1.pdf]

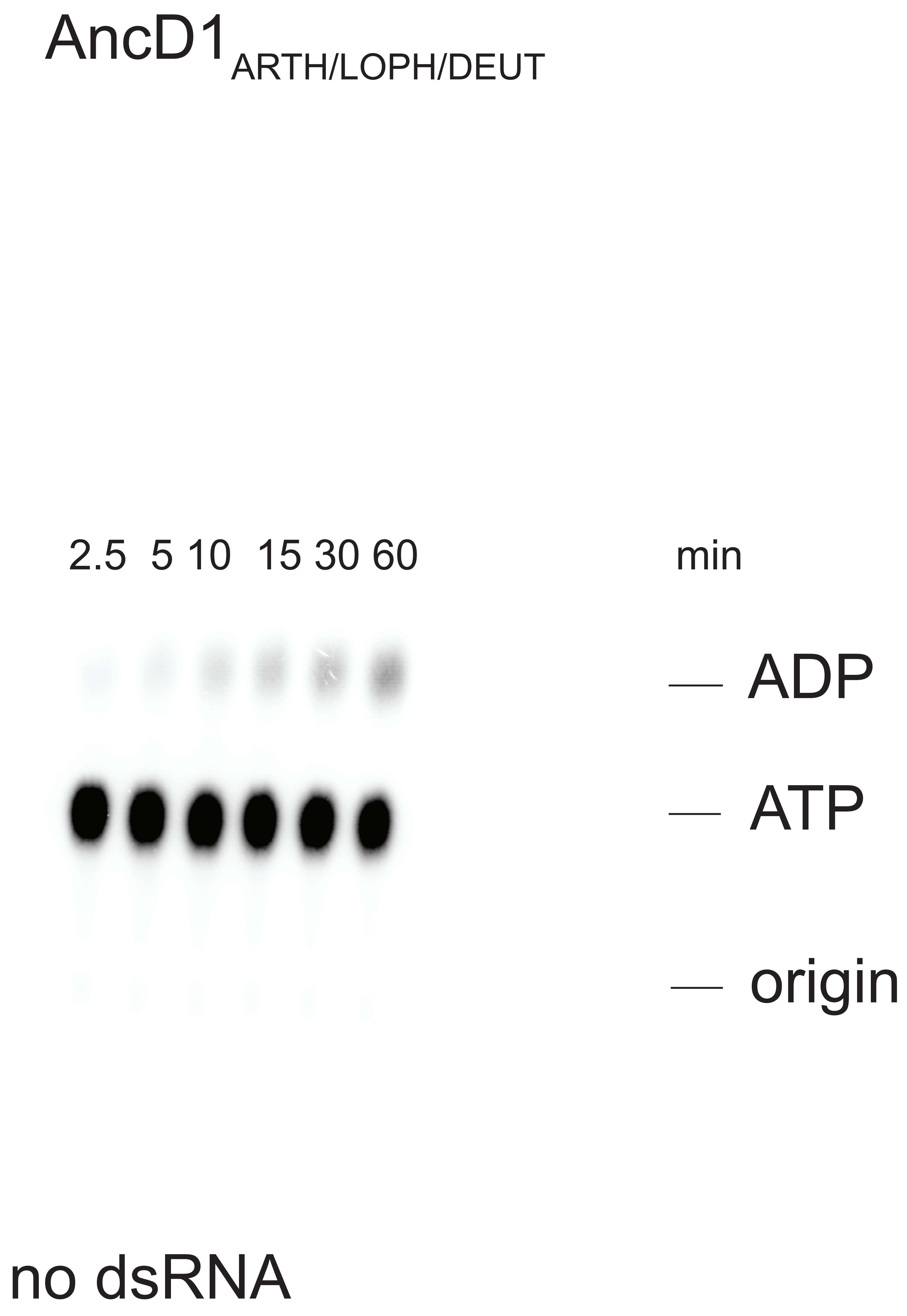


Figure 4-figure supplement 3 - source data 1: Original digital image of Thin Layer Chromatography plate used in A, left panel.
