# Supplementary material for: Ancestral protein reconstruction reveals evolutionary events governing variation in Dicer helicase function
Source: eLife. 2023 Apr 17;12:e85120. doi: 10.7554/eLife.85120 (PMC10159624; doi:10.7554/eLife.85120)
Supplement: Figure 4—figure supplement 3—source data 4. [file FIGURE 4-FIGURE SUPPLEMENT 3 - SOURCE DATA 4.pdf › FIGURE 4-FIGURE SUPPLEMENT 3 - SOURCE DATA 4.pdf]

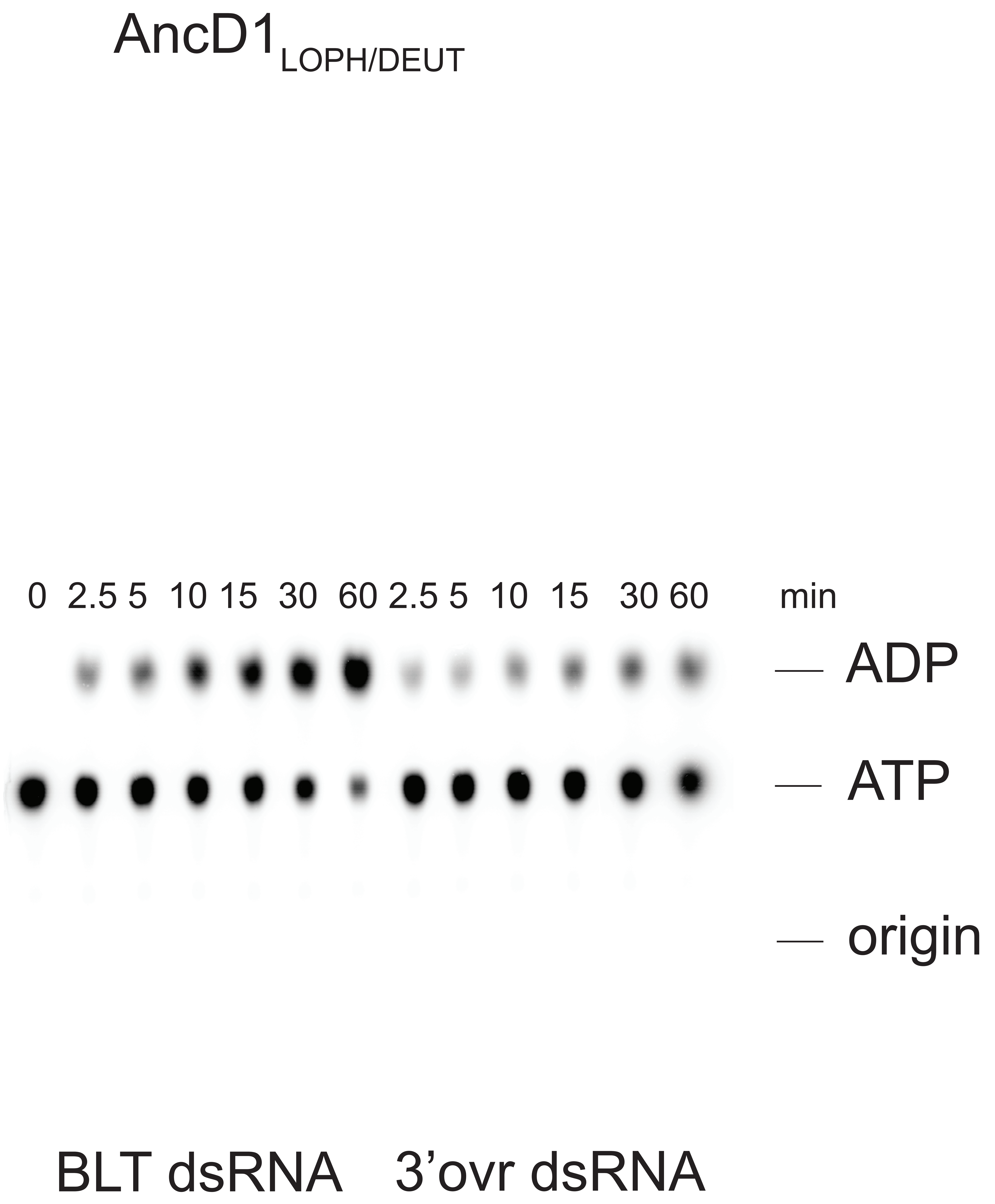

Figure 4-figure supplement 3 - source data 4: Original digital image of Thin Layer Chromatography plate used in B, right panel.
